# Unravelling the Metabolic Reconfiguration of the Post-Challenge Primed State in *Sorghum bicolor* Responding to *Colletotrichum sublineolum* Infection

**DOI:** 10.3390/metabo9100194

**Published:** 2019-09-20

**Authors:** Fidele Tugizimana, Paul A. Steenkamp, Lizelle A. Piater, Nico Labuschagne, Ian A. Dubery

**Affiliations:** 1 Research Centre for Plant Metabolomics, Department of Biochemistry, University of Johannesburg, Auckland Park, Johannesburg 2006, South Africa; ftugizimana@uj.ac.za (F.T.); psteenkamp@uj.ac.za (P.A.S.); lpiater@uj.ac.za (L.A.P.); 2 Department of Plant and Soil Science, University of Pretoria, Hatfield, Pretoria 0028, South Africa; nico.labuschagne@up.ac.za

**Keywords:** chemometrics, LC-MS, metabolomics, PGPR, plant priming, secondary metabolites, sorghum

## Abstract

Priming is a natural phenomenon that pre-conditions plants for enhanced defence against a wide range of pathogens. It represents a complementary strategy, or sustainable alternative that can provide protection against disease. However, a comprehensive functional and mechanistic understanding of the various layers of priming events is still limited. A non-targeted metabolomics approach was used to investigate metabolic changes in plant growth-promoting rhizobacteria (PGPR)-primed *Sorghum bicolor* seedlings infected with the anthracnose-causing fungal pathogen, *Colletotrichum sublineolum*, with a focus on the post-challenge primed state phase. At the 4-leaf growth stage, the plants were treated with a strain of *Paenibacillus alvei* at 10^8^ cfu mL^−1^. Following a 24 h PGPR application, the plants were inoculated with a *C. sublineolum* spore suspension (10^6^ spores mL^−1^), and the infection monitored over time: 1, 3, 5, 7 and 9 days post-inoculation. Non-infected plants served as negative controls. Intracellular metabolites from both inoculated and non-inoculated plants were extracted with 80% methanol-water. The extracts were chromatographically and spectrometrically analysed on an ultra-high performance liquid chromatography (UHPLC) system coupled to high-definition mass spectrometry. The acquired multidimensional data were processed to create data matrices for chemometric modelling. The computed models indicated time-related metabolic perturbations that reflect primed responses to the fungal infection. Evaluation of orthogonal projection to latent structure-discriminant analysis (OPLS-DA) loading shared and unique structures (SUS)-plots uncovered the differential stronger defence responses against the fungal infection observed in primed plants. These involved enhanced levels of amino acids (tyrosine, tryptophan), phytohormones (jasmonic acid and salicylic acid conjugates, and zeatin), and defence-related components of the lipidome. Furthermore, other defence responses in both naïve and primed plants were characterised by a complex mobilisation of phenolic compounds and *de novo* biosynthesis of the flavones, apigenin and luteolin and the 3-deoxyanthocyanidin phytoalexins, apigeninidin and luteolinidin, as well as some related conjugates.

## 1. Introduction

The interactions between plants and pathogens are complex and dynamic molecular battles, and the outcome is determined either by the successful establishment of the pathogen or by the efficiency of the host immune response mechanisms to ward off the infection [1]. In this co-evolving molecular arms race between pathogens and host plants, one of the central ontological mechanisms by which plants can increase their defensive efficiency and capacity is by pre-conditioning of the immune system through interactions with some signals or microorganisms in the environment—a phenomenon known as priming [2,3]. Plants, although autotrophic organisms, form associations with neighbouring plants, microflora and microfauna; and as sessile organisms, these interactions are fundamentally mediated and mostly achieved via chemical communication [4,5]. Thus, the phenomenology of priming, known as sensitisation as early as 1933, results from the interactions between the host plants and beneficial habitants of the rhizosphere (rhizobacteria, mycorrhizal fungi), virulent or avirulent pathogens, or by natural or synthetic compounds that include some agrochemicals [2,3,5].

Mechanistically, priming can be described as a spatially and temporally complex, multistage process at cellular and molecular levels. The progression consists of three main stages namely: the priming phase, the post-challenge primed state and the transgenerational primed state [3,6]. Although detailed molecular mechanisms still remain elusive, studies have proven priming to be a key process in various forms of systemic plant immunity [2]. Such defence-priming comprises (i) systemic acquired resistance (SAR), which is induced by necrotising pathogens and requires salicylic acid (SA), pipecolic acid (PA), dehydroabietinal (DA) and azelaic acid (AzA) [7]; (ii) induced systemic resistance (ISR), activated by mutualists such as plant growth-promoting bacteria (PGPR) and fungi in the rhizosphere, and orchestrated by jasmonate (JA)- and ethylene (ET)-dependent mechanistic paths [2]; (iii) wound-induced resistance [8]; and (iv) β-aminobutyric acid-induced resistance (BABA-IR) [9]. The rhizobacteria-mediated ISR has received increasing attention in current research trends. Detailed accounts of the complexity of the rhizosphere, its dense and diverse microbiome population and molecular signalling web have been published [10,11]. Although the rhizosphere chemistry remains largely unknown, and the establishment of plant-rhizomicrobiome mutualistic interactions is still poorly characterised, emerging studies have reported that various PGPR species of the genera *Pseudomonas*, *Bacillus* or *Bradyrhizobium*, and the plant growth-promoting fungi (PGPF) from the *Trichoderma* genus, prime the whole plant for increased defence preparedness against a broad range of both below- and above-ground pathogens and attackers [3,5,10]. The molecular mechanisms underlying the rhizobacteria-related defence priming indicate that this induced state implies reprogramming of cellular metabolism and regulatory machinery [3,5,6].

Current insights propose that, preceding infection, primed plants re-organise supporting metabolic pathways by modifying the biosynthesis of sugars, tricarboxylic acids and amino acids [12], in preparation for the activation of chemical defences based on secondary metabolism [13]. However, the current knowledge of biochemical and molecular mechanisms of defence priming is just a tip of an iceberg, and a detailed mechanistic and functional description of the various layers of priming events is still limited. Biochemical and molecular networks driving successful establishment of priming phases are still far from being completely elucidated and predictively characterised. Nonetheless, despite these limitations (and many more uncertainties and grey areas, with regard to spatial and temporal mechanisms of priming), this potentiation of the immune system is undoubtedly a fundamental means that plants have evolved as an adaptive strategy: by immunologically memorising a stress so as to amplify defensive capacity upon subsequent stresses. As such, defence priming represents a promising and complementary alternative strategy that can provide new opportunities for plant protection against pathogens.

*Sorghum bicolor* is an important grain crop for human nutrition, phytochemical neutraceuticals and biofuel usage [13]. We have previously reported on the metabolomics of defence-related reprogramming in sorghum plants in response to infection by the hemibiotrophic pathogen, *Colletotrichum sublineolum.* Results revealed synchronised activation of a functional metabolic web of secondary metabolites originating from the phenylpropanoid—and flavonoid pathways [14]. In the present study, an untargeted liquid chromatography coupled to mass spectrometry (LC-MS) metabolomic approach was employed to elucidate metabolic profiles underlying the enhancement of sorghum defence responses when primed/pre-conditioned by a rhizobacterium, *Paenibacillus alvei.* In a companion study we have recently reported on the effectiveness of the *P. alvei* (strain T22) as a bacterial PGPR priming agent in the sorghum: *Fusarium pseudograminearum* pathosystem [15]. Thus, the current study was designed to focus on the post-challenge primed state to unravel metabolic/molecular reconfiguration of the sorghum primed metabolism in response to *C. sublineolum* infection. Considering that the metabolome is more sensitive to alterations in both metabolic fluxes and enzyme activity than either the transcriptome or proteome [16,17], the measurement of dynamic changes of the metabolites would thus informatively reflect differential and functional features of the primed metabolism. Such endeavour would pinpoint metabolic pathways involved in the rhizobacteria-induced systemic resistance in sorghum against fungal infection, thus contributing to the increasing attempts to unravel the biochemical and molecular mechanisms involved in defence priming.

## 2. Results and Discussion

### 2.1. Evaluation of Anthracnose Symptom Development in P. alvei Primed vs. Naïve Sorghum Plants Challenged with the Hemibiotrophic Pathogen, Colletotrichum sublineolum

The disease phenotype assessment showed that the sorghum plants primed with *P. alvei* strain T22 were more resistant to *C. sublineolum* infection. Following pathogen challenge, the primed sorghum plants showed only slight symptom development and only starting at 6 days post infection (d.p.i.) (Figure S1). The anthracnose symptoms that appeared on the primed sorghum plants following the fungal pathogen challenge were significantly less severe, even at 9 d.p.i., compared to the non-primed challenged plants: few leaves and plants showed symptoms, which could even be seen as localised hypersensitive response (HR) lesions, with no spreading over the entire leaf surface (Figure S1A). The beneficial rhizobacterium, *P. alvei*, has previously been shown to be an effective plant growth-promoting and biocontrol agent in wheat and tomato plants [18,19] and the results from the disease phenotype assessment (Figure S1B) clearly suggest that this PGPR also primed the sorghum plants and conferred an evidently effective resistance against *C. sublineolum*. Thus, to gain an inclusive overview of the metabolic reprogramming associated with chemical defences related to the priming effects of *P. alvei* (T22), an untargeted LC-MS-based metabolomic analysis was performed. The focus was on the metabolic changes as reflected in hydromethanolic extracts of sorghum seedlings during the post-challenge primed stage.

### 2.2. Comparative Analysis of the Metabolomic Profiles of P. alvei (T22)-Primed and Naïve Sorghum Plants Challenged with the Anthracnose Pathogen, Colletotrichum sublineolum

**Figure 1:**
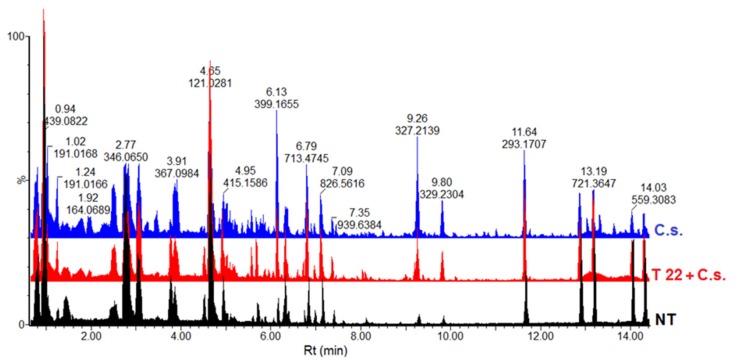
Representative UHPLC-MS chromatograms of ESI(-) data (5 d.p.i.). Base peak intensity (BPI) mass chromatograms displaying comparative chromatographic differences in different conditions: (i) samples from non-treated plants (NT), (ii) samples from *P. alvei* (T22)-primed and *C. sublineolum* (*C.s*.)-challenged plants and (iii) samples from *C.s.*-infected plants. Visual inspection of the chromatograms evidently shows differential peak populations, for instance in the 4–12 min retention time (Rt) region.

From a metabolism perspective, the metabolic shift, induced by priming effects of the *P. alvei* PGPR on sorghum seedlings responding to fungal infection, may span several pathways. Hence, an untargeted approach was opted for in this study to allow inclusive coverage of the metabolome; however, at the same time, acknowledging the current metabolomics bottlenecks that collectively (or separately) make it realistically impossible to achieve a truly comprehensive metabolic picture. Thus, using an UHPLC connected in-line with a high-definition MS instrument, and acquiring data in both positive and negative electrospray ionisation (ESI+/−) modes, allowed a non-biased, global analysis and detection of semi-polar to nonpolar metabolites extracted with 80% methanol. Considering the axiomatic complexity and multidimensionality of the metabolome, good chromatographic separation was achieved (e.g., Figure 1), which is an essential preceding step before MS analyses for enhanced signal sensitivity [16]. Furthermore, differential metabolic profiles were visually observed, as indicated by base peak intensity (BPI) mass chromatograms (e.g., Figure 1 and Figure S2) and pointing to metabolic changes related to the priming effects of *P. alvei* (T22) on sorghum plants responding to *C. sublineolum* infection.

**Figure 2:**
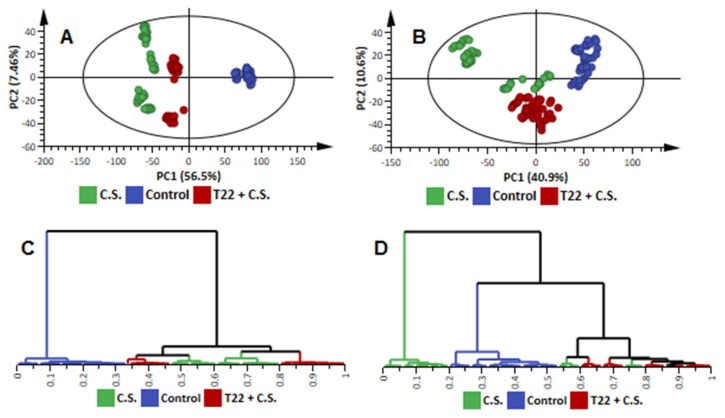
Exploratory data analysis with unsupervised chemometric methods. (**A**): A PCA scores scatter plot computed from the first two PCs of a model of ESI(+) data of all time points combined. The data matrix is presented as an eleven-component (R1 significance) model, explaining 79.5% variation in the Pareto-scaled data and the amount of predicted variation by the model, according to cross-validation, is 72.7%. (**B**): A PCA scores scatter plot computed from the first two PCs of a model of ESI(-) data, X: an eleven-component (R1 significance) model, explaining 73.6% variation in the Pareto-scaled data and the amount of predicted variation by the model, according to cross-validation, is 65.9%. (**C**,**D**) are HCA dendrograms corresponding to (**A**,**B**), respectively. The ellipse in the PCA scores plots represents the multivariate generalisation of the 95% confidence interval (Hotellings T^2^), the normality area, which is used to visually identify strong outliers. Corresponding data from the ESI(-) mode, indicating the different time points, is shown in Figure S3.

Although these chromatographic fingerprints (Figure 1 and Figure S2) provided a visual portrayal of metabolic differences between samples from different conditions (e.g., challenged primed plants vs. naïve plants), informative details about metabolite features/structures underlying the metabolic reprogramming related to *P. alvei* (T22) priming effects can only be achieved through data mining and comparative chemometric analyses. An unsupervised bilinear factor modelling, principal components analysis (PCA), was performed for exploratory analyses of the collected metabolomic data: to summarise the multidimensional data in an intelligible way (reduced dimensional space) that grasps the silent characteristics of the data [16,20]. The computed PCA models, with no overfitting (Figure 2), allowed descriptive evaluation of the distribution of samples so as to detect natural groupings, trends and outliers. The graphical visualisation generated by these models yielded a natural separation/clustering in the scores space between samples from (i) sorghum plants that were neither treated with fungal pathogen nor *P. alvei* (T22) (control), (ii) naïve plants treated with *C. sublineolum* (*C.s.*), and (iii) *P. alvei* (T22)-primed plants challenged with the pathogen (T22 + *C.s.*) (Figure 2A,B and Figure S3). Previously, a companion study using identical extraction methods (80% methanol) and analytical conditions (C_18_ reverse phase chromatography), indicated no significant differences in the metabolic profiles as reflected in PCA scores plots of extracts from control plants and that of *P. alvei*-treated plants [15]; the latter condition was therefore not included in the experimental planning and further analysis.

In both ESI(−/+) modes, three clear major sample groupings related to the three conditions mentioned (control, *C.s*.-inoculated plants and *C.s*.-challenged primed plants) are evidently seen in the scores space (Figure 2A,B), thus reflecting differential metabolic features in those three groups. The observed variation within groups is related to the time points or metabolite collection time, i.e., 1–9 d.p.i., where early responses in either naïve or primed plants are closer to each other (Figure S3D). However, in the PCA space, this time-related variation appears to be less pronounced compared to the major variation induced by the treatment (fungal infection of primed vs. naïve plants, Figure 2A,B). Furthermore, the responses to the fungal infection in both naïve and primed plants were clearly characterised by a significant metabolic shift, visualised in the PCA scores plots as the samples of the two conditions cluster separately from the control. PC analyses also showed that the samples from the infected primed plants grouped differentially from the infected naïve plants, with no overlap between the two groups (Figure 2A,B). This points to underlying different metabolic states related to defence mechanisms against the fungal infection between naïve and *P. alvei* (T22)-primed plants.

In order to further investigate these PCA-described natural groupings in the metabolite (variable) space, rather than the sample space, an unsupervised multivariate method, hierarchical cluster analysis (HCA), was applied to the PCA-transformed (low-dimensional) data. As a clustering method, HCA is based on the multivariate distance between pairs of data points—in this case, metabolite information in each sample is expressed by a vector. This approach allows to compute, in series, clusters that show multivariate similarity in the variable (metabolite) space [21]. Thus, agglomerative HCA models were computed using Euclidean distance and Ward’s minimum variance as a dissimilarity and linkage rule, respectively. The generated hierarchy of clusters, displayed as dendrograms, were evaluated (Figure 2C,D). Two distinct major clusters were observed in the ESI(+) data, corresponding to control samples (blue line) differentially separated from the fungal treated (naïve and primed) samples (Figure 2C). For the ESI(-) mode data, although control samples clustered different from treated samples, the data from treated naïve plants (5–9 d.p.i.) formed a major cluster (green line, Figure 2D). In both ESI modes, several time-related distinct subspaces in each major cluster were observed (Figure 2C,D). Although HCA does not readily identify which metabolite features are responsible for the observed sample classifications, the generated hierarchical clusters pointed to inner structures of the data (in the metabolite space), confirming the observations from PCA results (Figure 2A,B), and further pointing to underlying differential (dynamic) metabolism of naïve and *P. alvei* (T22)-primed sorghum plants in response to fungal infection (Figure 2C,D). Both these explorative (unsupervised) data analyses, i.e., PCA and HCA models, permitted a descriptive overview of the data, and allowed for extracting informative sample distribution patterns and groupings. As captured by these models, the analysed metabolite features (in both ESI modes) clearly differentiated the defence responses of *P. alvei* (T22)-primed from naïve sorghum plants (Figure 2 and Figure S3) and suggests differential defence mechanisms against the *C. sublineolum* infection. Such extrapolation (supported also by the disease phenotype assessment—Figure S1) would correlate to the view that in the face of a secondary stress challenge, primed plants deploy faster and stronger defence responses compared to naïve counterparts, thus reflecting differential underlying metabolism [3,6].

**Figure 3:**
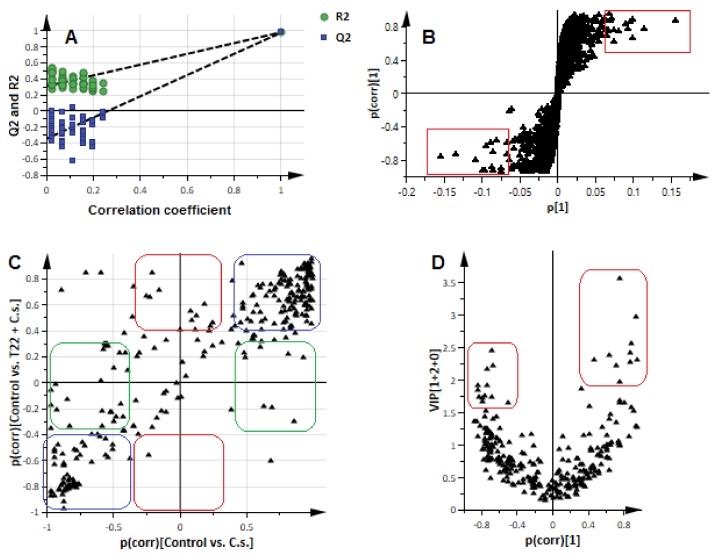
OPLS-DA modelling and variable (statistically significant discriminatory features/biomarkers) selection. (**A**): A typical response permutation test plot (*n* = 50) for the OPLS-DA model of the ESI(-) data, separating ‘control vs. challenged primed-plants’ at 5–9 d.p.i. (1 + 2 + 0 components, R^2^X = 0.658, Q^2^ = 0.978, CV-ANOVA *p*-value = 0.00); the R^2^ and Q^2^ values of the permutated models correspond to *y*-axis intercepts: R^2^ = (0.0, 0.308) and Q^2^ = (0.0, −0.347). (**B**): An OPLS-DA loadings S-plot for the same model in (**A**); variables situated far out in the S-plot are statistically relevant and represent prime candidate discriminating variables. The red boxes indicate the selected ions—as discriminating variables, with high covariation and correlation. (**C**): A Shared and Unique Structures (SUS) analysis: a SUS-plot comparing the metabolite features contributing to the model separating ‘control vs. challenged naïve plants’ (control vs. *C.s*., ESI(-) data, *x*-axis; 1 + 1 + 0 components; R^2^X = 0.704, Q^2^ = 0.993, CV-ANOVA *p*-value = 0.00) with that of the model separating ‘control vs. challenged primed plants’ (control vs. *P. alvei* (T22)+*C.s*., ESI(-) data, *y*-axis; 1 + 2 + 0 components, R^2^X = 0.658, Q^2^ = 0.978, CV-ANOVA *p*-value = 0.00). Blue boxes represent shared features, green boxes represent features linked with the response of naïve plants to the fungal infection and red boxes the unique features due to the combination of priming and fungal infection. (**D**): A V-like shaped plot (Plot/List Plot) displaying the combination of variable importance in projection (VIP) scores and p(corr) values for the variables in S-plot in (**B**).

For biochemical characterisation and interpretation of these informative metabolite profiles described by explorative modelling (Figure 2), a supervised method namely ‘orthogonal projection to latent structures-discriminant analysis’ (OPLS-DA) was applied. OPLS-DA allows the identification of the metabolite features/markers underlying the discrimination between classes or groups [16,22]. The computed and validated OPLS-DA models (CV-ANOVA *p*-value ˂ 0.05) used were perfect binary classifiers. No signs of possible overfitting, as indicated by cross-validation, were found and none of the permutated models (*n* = 100) performed better than the original models in separating classes (Figure 3A and Figure S4). OPLS-DA loadings plots were thus evaluated for selection of discriminating metabolite features/variables with unique retention time (Rt) and *m*/*z* values (Figure 3B,D).

The selection of the variables was carried out with mathematical/statistical rigour, avoiding selection bias or any false positives. An inherent high degree of inter-connectivity between metabolites in a biological network, accentuated with untargeted analysis, necessitates the application of multivariate variable selection methodology. As such, correlation and co-variance, confidence interval and orthogonal variation, rather than *t*-statistics, is the first statistically suitable step in extracting and identifying discriminating features/potential biomarkers [23]. Thus, evaluation of the OPLS-DA loadings S-plots (Figure 3B) allowed the identification of statistically significant potential discriminant features, i.e., variables that combined both high correlation and covariation [16,24].

Furthermore, considering the design of this study, investigating simultaneously different conditions (naïve and primed sorghum plants, both responding to a fungal infection), and the sample distribution in the multivariate (sample and metabolite) space as revealed by unsupervised modelling (e.g., Figure 2), another variable selection method, namely the ‘Shared and Unique Structures’ (SUS) loadings plot was applied (Figure 3C). The SUS-plot displays shared and unique features between two different classification models that have a common reference: the correlation from the predictive component, Corr(*t*_p_,*X*), of each model is plotted against each other, allowing the identification of shared and unique variables [24]. For instance, Figure 3C is a SUS-plot comparing the metabolite features from two OPLS-DA models, in this case ESI(-) data of control vs. challenged naïve plants (*x*-axis) and control vs. challenged primed plants (*y*-axis). Given that both models have the same baseline point (control), the differential metabolites between naïve- vs. primed-plants (both responding to the fungal infection) are extracted in the plot. The metabolite features that are of equal importance for the two models cluster along the (latent) diagonal (blue boxes—bottom left and upper right corners) and are of little use as biomarkers as they represent shared features. The metabolite features altered only due to the combination of priming and fungal infection are located along the *y*-axis (red boxes) and represent unique structures. In comparison, the variables located along the *x*-axis (green boxes) are unique features that characterise the response of naïve plants to the fungal infection.

Thus, both OPLS-DA S-plots and SUS-plots (Figure 3B,C and Figure S4) allowed the identification of subsets of discriminating variables that explain the differential metabolite profiles described by explorative modelling (Figure 2). These prime candidates or potential biomarkers were further evaluated and validated for statistical significance and performance. Different tests were applied with the Variable Importance/Influence in Projection (VIP) > 1.0 as the initial validation check-point (Figure S4B). VIP scoring is a metric that summarises the importance of each variable in driving the observed group separation in a classification modelling, and a variable with a VIP score > 1.0 indicates that it contributes more than average to the model, hence its relevance and statistical basis for its selection [25].

However, since the VIP scoring changes with each iteration of variable selection, complementing VIP values with stable parameters such as the p(corr) values provides a robust mathematical framework to confidently evaluate the performance and reliability of candidate variables [26,27,28,29]. Hence, in this study, the combination of VIP scores and p(corr) values was used and plotted (Figure 3D). Such a V-like shaped plot enables the identification of metabolite features with high (separation) importance to the model (VIP score > 1.0) and high predictability (high absolute p(corr) values)—these variables are located in upper regions of the plot (Figure 3D). Additional assessment methods for variable selection included ‘jackknife’ confidence intervals, variable trends, dot plots and descriptive statistics (Figure S4D) [25,30] as well as manually checking the signal stability of the candidate features in the QC samples. Thus, the selected and validated metabolite features (markers explaining the metabolic alterations revealed by PCA and HCA models) were then annotated to the Metabolomics Standards Initiative (MSI-level 2 annotation), as described under Materials and Methods and reported in Table 1. Annotated metabolites are reported together with their respective fold-changes (ratios of peak intensities in extracts from infected primed plants compared to the infected naïve, non-primed counterparts) for the post-challenge periods of 1–3 d.p.i. and 5–9 d.p.i. Fragmentation data of the metabolites is presented in the accompanying Table S1.

### 2.3. Differential Defence-Related Metabolic Changes in P. alvei Primed vs. Naïve Sorghum Plants Challenged with the Hemibiotrophic Pathogen, Colletotrichum sublineolum

As highlighted in the introductory section, the horizontal phenomenon of priming is to potentiate the plant immune system so as to place the plant in an alert state for strong, rapid and effective defence responses upon subsequent or secondary stresses [6], such as pathogenic fungal infections as is the case of this study. This implies that a primed plant wards off a pathogenic invasion more efficiently than a naïve plant, which indicates a differential defence metabolism. Thus, for a biochemical description of the metabolic changes revealed by chemometric analyses, i.e., a dynamic defence metabolism differentiating naïve—and primed sorghum plants challenged with the pathogen *C. sublineolum*, the annotated metabolites extracted from the S-plots and SUS-plots (Table 1) were quantitatively assessed. These chemometrically identified and most relevant compounds in the post-challenged primed stage spanned several metabolic pathways; including primary metabolism (amino acid pathways), phytohormones, lipid metabolism and secondary metabolism (phenylpropanoid—and flavonoid biosynthesis pathways) (Table 1).

#### 2.3.1. Differential Changes in Primary Metabolism and Plant Hormones Levels

**Figure 4:**
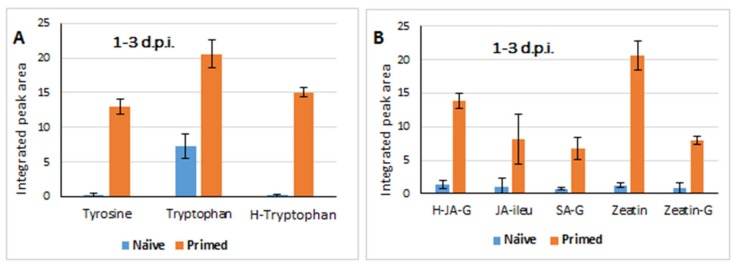
Quantitative profiles of amino acids and phytohormones in samples from both naïve and primed plants responding to the *Colletotrichum sublineolum* fungal infection. The integrated peak area of each compound (extracted from MarkerLynx-XS^TM^-based processing) over the time period of 1–3 d.p.i. was used for relative quantitation of the levels of the metabolites. Data represent the average of three experiments (with three replicates in each experiment), *n* = 45 and *p*-values reported as in Table 1; error bars = standard deviation. (**A**): Amino acid profiles and (**B**): Plant hormones profiles. H-JA-G = hydroxyjasmonic acid-glucoside; JA-ileu = jasmonoyl-isoleucine; SA-G = salicylate-glucoside; zeatin-G = *trans*-zeatin-glucoside (Table 1).

The evaluation of the SUS-plots (simultaneous, multivariate assessments of the responses of both naïve—and primed plants to the fungal infection) allowed the identification of unique metabolite features that differentiate the naïve vs. primed defence responses. Some of the observed predominant differences include the changes in amino acids and phytohormones in the early phase of the fungal infection (1–3 d.p.i.). The evaluation of the metabolic changes, following fungal infection showed that the primed plants significantly accumulated tyrosine, tryptophan and hydroxytryptophan compared to the infected naïve plants. The level of these metabolites remained high for the period of 1–3 d post-secondary challenge (fungal infection) (Figure 4A).

Previous studies have reported that one of the characteristics of the priming phase involves alterations in primary metabolism including the accumulation of amino acids, which are then channelled into different defence-related metabolic pathways when the primed plants are challenged [12,31]. Furthermore, a new understanding of the regulation of plant immunity by amino acid metabolic pathways is emerging, pointing to different mechanistic roles of amino acids in plant defence responses to pathogen attack [32,33]. The perturbance/enhancement of the amino acid pools in the priming phase corresponds with the pre-conditioning of the plants, and upon the subsequent secondary challenge (fungal infection in this case), the deployment of these amino acids is rapid and more intense compared to the response in the naïve plants as the relative quantitative profiles indicate in this study (Figure 4A). In addition to being essential components of synthesis of new defence proteins, the aromatic amino acids tryptophan, phenylalanine and tyrosine serve as precursors for a wide range of secondary metabolites that are functionally important for plant interactions with the environment such as deployment of anti-microbial phytoalexins [34,35].

Although the chemical nature and mechanistic details of tryptophan-derived metabolites remain elusive in some cases, experimental evidence indicate that these compounds play crucial roles in plant immune responses to a wide range of pathogens [12,36]. Tryptophan metabolism has been shown to be involved in the defence responses of rice with tryptamine, 5-hydroxytryptophan and serotonin increasing markedly in *Bipolaris oryzae*-infected leaves for 72 h after inoculation [37]. Tryptophan-derived secondary metabolites have also been identified to play a central role in defence responses of Arabidopsis against necrotrophic *Plectosphaerella cucumerina* infection by limiting the growth of this fungal pathogen, and against the vascular fungal pathogen, *Verticillium longisporum* [36,38]. The extent of activation of tryptophan-derived metabolites and utilisation thereof in a defence-related context was shown in a detailed metabolomics study of Arabidopsis responding to bacterial lipopolysaccharides where a number of indole derivatives, camalexin and indole glucosinolates were reported as biomarkers [39]. Furthermore, as mentioned above, this primary metabolic reconfiguration also implicated a significant increase in tyrosine levels in naïve- vs. primed plants, in response to *C. sublineolum* infection (Figure 4A). Defence-related accumulation of tyrosine has previously been reported in different phythopathosystems [40,41], highlighting its functional role in plant immune responses. Tyrosine is an upstream precursor of the plant defence cyanogenic glucoside, dhurrin [42,43], which was found in this study to be significantly higher (relatively 40× and 24×, in the post-challenge periods of 1–3 d.p.i. and 5–9 d.p.i, respectively, (Table 1) in primed plants compared to the naïve counterparts.

Chemometric analyses and relative quantitation also revealed a significant accumulation of plant hormones (zeatin and/or the glycosylated forms such as zeatin-glucoside, salicylate-glucoside and hydroxyjasmonic acid glucoside) as a differentiating characteristic of the defence responses of primed plants (Figure 4B and Table 1). Phytohormones coordinate multiple physiological and biochemical processes in plants via the regulation of gene expression. These include control of growth and development, regulation of cellular activities such as cell division and differentiation, organogenesis, and importantly, responses to abiotic and biotic stresses [44,45,46]. The elaborate web of crosstalk between various phytohormones, such as defence signalling hormones (e.g., salicylic acid and jasmonates) and growth regulators (e.g., cytokinins), either through synergistic or antagonistic interactions, plays a crucial role in fine-tuning trade-off between growth and defence in plants [47,48]. Most studies on the role of phytohormones in plant-pathogen interactions have focused on salicylic acid (SA), jasmonic acid (JA), abscisic acid (ABA) and ethylene (ET), elucidating and describing underlying regulatory and signalling mechanisms of these hormones and their essentiality in plant responses to biotic stresses. The topic has been extensively reviewed [49,50], however, it suffices to point out that JAs coordinate the ISR-response activated by mutualists such as PGPR and fungi in the rhizosphere [51,52]. Thus, the observed higher levels of glycosylated JA and JA-ileu (Figure 4B) in the *P. alvei*-treated plants (compared to naïve plants) responding to the fungal infection confirm the primed state thereof, and conferred readiness for a stronger defence response to the *C. sublineolum* infection.

Furthermore, the cytokinin (CK), zeatin, and its glycosylated form (*trans*-zeatin-glucoside) were found to be unique features (exhibiting increased levels) differentiating the responses of naïve- and primed plants to fungal inoculation (Figure 4B and Table 1). The physiological roles and functional mechanisms of altered CK homeostasis in stress responses are still largely unclear. Most of the studies on CKs have been done in model systems such as *Arabidopsis thaliana* [53,54], hence, the current limited understanding and knowledge about the dynamics and regulatory mechanisms of these molecules in crop plant-pathogen interactions. CKs are an important class of phytohormones affecting numerous developmental processes (such as cell proliferation and differentiation, nutrient status and circadian clocks), and zeatin and its derivatives have been reported to be the most important group of isoprenoid CKs [55,56]. Recent reports have shown that the zeatin-type CKs enhance resistance against pathogens in Arabidopsis and tobacco plants, associated with increased cell membrane integrity. Transgenic Arabidopsis plants with stabilised high levels of CKs exhibited increased resistance against infection by the hemibiotrophic pathogen *Verticillium longisporum* [57]. CKs have also been shown to regulate stomatal conductance of wheat seedlings, pointing to the role of these hormonal molecules in plant-environment interactions [58]. Furthermore, the activation of (zeatin-type) CK signalling has been observed in Arabidopsis plants primed with a PGPR, *B. subtilis* [59]. Although the role of CKs in plant immune responses is still poorly understood, these emerging experimental evidences indicate that CKs are involved in the regulation of plant defences against a wide range of pathogens, often via crosstalk with SA or JA [48,60].

Thus, these quantitative observations, namely (i) higher levels of amino acids, tyrosine, tryptophan, 5-hydroxytryptophan (Figure 4A) and their downstream derivatives (indole-acetyl conjugates, and defence-related secondary metabolites, namely dhurrin and feruloyl-serotonin, Table 1), and (ii) higher levels of zeatin (and its glycosylated derivative) as well as the increased levels of JA- and SA-related compounds (Figure 4B and Table 1), evidently reflects a stronger and more efficient defence response deployed by primed sorghum plants against infection by *C. sublineolum*. It can thus be correctly postulated that the priming of the sorghum plants by rhizobacterium *P. alvei* (T22), potentiated the plants to rapidly launch defence mechanisms, involving reprogramming of primary metabolite pools and a web of crosstalk between regulatory pathways, for immediate strong reactions (as early as 1–3 d.p.i.) to block the fungal invasion and further proliferation.

Plant-pathogen interactions are highly complex chemical battles, where both the host and pathogen vie for sustainable survival, and the outcome is determined by the directional shift of these chemical communications [5]. This may be either towards successful proliferation of the pathogen or effective immune responses of the host. Furthermore, the establishment and maintenance of the biotrophic stage (which is about 72 h post-infection) is crucial for successful development of the hemibiotrophic *Colletotrichum* spp., and for an effective infection thereafter [61,62,63]. Thus, this strong defence-related metabolic reprogramming observed in primed plants (compared to naïve plants) as early as 1–3 d.p.i. (Figure 4 and Table 1), pinpoints the molecular preparedness and impetus of the primed plants to quickly halt the successful invasion and establishment of a *C. sublineolum* infection. This postulation is also supported by the symptomatology results: no significant anthracnose disease development was observed in the primed plants even in the later stage of infection period (Figure S1).

#### 2.3.2. Defence Responses in Colletotrichum sublineolum-Challenged (Primed vs. Naïve) Sorghum Plants: Differential Changes in the Lipidome and Phenolics

**Figure 5:**
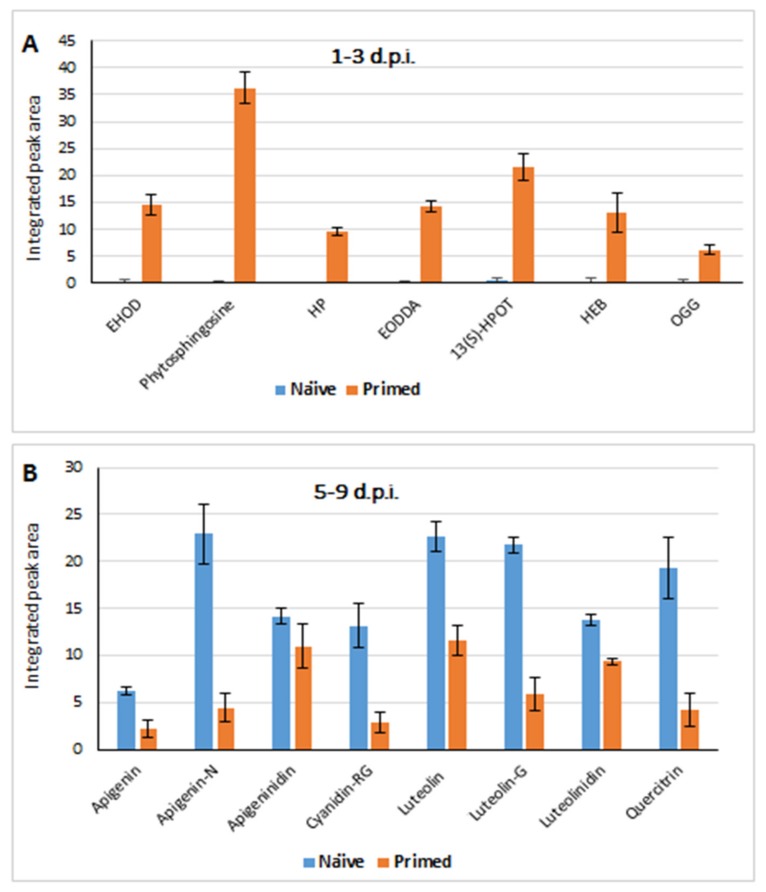
Quantitative profiles of lipids and flavonoid metabolites in samples from both naïve and primed plants responding to the *Colletotrichum sublineolum* infection. (**A**): Lipid profiles; EHOD = epoxy-9-hydroxy-10-octadecenoate; HP = 16-hydroxypalmitate; EODDA = epoxyoctadeca-9-11-dienoic acid; HEB = hydroxy-24-epi-brassinolide; OGG = oleanolate-glucoronoside-28-glucoside. (**B**): Flavonoid profiles; apigenin-N = apigenin 7-O-neohesperidoside; cyanidin-RG = cyanidin 3-O-rhamnosylglucoside; luteolin-G = luteolin 7-O-glucoside. In both (**A**) and (**B**) data represent the average of three experiments, *n* = 45 with *p*-values reported in Table 1. The integrated peak area of each compound (extracted from MarkerLynx-XS^TM^-based processing) over the time period of 1–3 d.p.i. (**A**) and 5–9 d.p.i. (**B**) were used.

The relative quantitative evaluation of the chemometrically-derived biomarkers revealed a significant reprogramming of the lipidome as another characteristic of primed plant responses to the fungal infection. Following *C. sublineolum* inoculation (1–3 d.p.i.), a number of the lipidome components (such as hydroxypalmitate, epoxy-hydroxy-octadecenoate and phytosphingosine) were found to be significantly accumulated in *P. alvei*-primed plants compared to naïve sorghum plants (Figure 5A and Table 1). Generally the plant lipidome is structurally diverse, comprising fatty acids, glycolipids, phospholipids, sphingolipids, sterol lipids and waxes [64,65]. In addition to being structural components of the plasma—and intracellular membranes, lipidome components are involved in diverse biological functions including storage of carbon and energy, signal transduction and stress responses [65,66].

Fatty acids and hydroxy fatty acids, such as those identified in this study namely hydroxypalmitate and epoxy-hydroxy-octadecenoate (Figure 5A and Table 1), have been found to be resistance-related compounds that particularly prevent pathogen penetration and proliferation by strengthening cell wall and membrane [65,67]. These fatty acids and hydroxy derivatives are some of the major constituents of cutin and waxy polyester matrices, forming a protecting film that controls the fluxes of gases and water, and prevents easy entry of harmful substances and pathogens into the host [67,68]. On the other hand, the 4-phytosphingosine (t18:0, also found to be increased in this study—Figure 5A) is one of the plant sphingolipids and that are initially essential membrane components [69,70]. An increased level of free phytosphingosine has been observed in *A. thaliana* infected with *Pseudomonas syringae*, pointing to a positive role of t18:0 in the defence responses to pathogens [71]. Experimental evidences have also indicated regulatory effects of phytosphingosisne-1-phosphate on stomatal aperture in Arabidopsis plants [72]. Furthermore, the sphingolipid content has been shown to modulate defence responses in Arabidopsis plants infected with hemibiotrophic and necrotrophic pathogens [73].

Thus, the elevated levels of these lipid components in primed plants vs. naïve plants, particularly hydroxy fatty acids and phytosphingosine (Figure 5A and Table 1) which have been reported to be resistance-related compounds, further confirm the preconditioning effect of *P. alvei* (T22) on the immune system of sorghum plants. Another lipid component that was found to be increased in the primed plants challenged with *C. sublineolum* was 13(S)-hydroxyperoxyoctadecatrienoic acid (13(S)-HPOT, Figure 5A). This metabolite is one of the essential intermediates in the biosynthetic pathway of JA. The first step of JA biosynthesis begins in the chloroplast membrane, with lipases cleaving lipids to generate linolenic acid, which is then oxygenated by 13-lipoxygenases to form 13(S)-HPOT. The latter is a substrate of multiple downstream enzymatic reaction steps that lead to the formation of JA [66,74]. Given that JA is an essential regulatory signalling molecule in PGPR-induced ISR, the higher level of 13(S)-HPOT in primed plants than in non-primed (Figure 5A) also corroborates the strong and rapid defence responses in primed sorghum against *C. sublineolum*. In addition to these metabolic reconfigurations that significantly differentiated the deployed defence mechanisms of primed compared to naïve plants, a reprogramming of phenolic profiles was also observed in both naïve and primed plants as a response to the fungal infection (Figure 5B and Table 1). The major components of this accumulated complex of phenolic compounds were the 3-deoxyanthocyanidin phytoalexins (luteolinidin, apigeninidin), luteolin and apigenin and some of the corresponding conjugates (Figure 5B and Table 1).

However, the quantitative analyses indicate that the levels of these defence-related flavonoids (particularly phytoalexins) were relatively higher as a result of the infection in naïve plants compared to primed plants (Figure 5B). This could be explained by the fact that the latter showed an early stronger response (as inferred from Figure 4 and Figure 5A) that might negatively impact on the biotrophic establishment of the fungus—i.e., a quicker and stronger blocking of pathogen penetration and invasion, and hence a less quantitative deployment of phytoalexins. Furthermore, the released active forms of these phytoalexins are toxic to both the fungus and plant cells that synthesised them [75,76]. It is therefore possible that the economy and logic of survival would dictate the less costly option, if available, and the release of toxins when necessary to be the last resort. However, targeted and absolute quantitative methods of these phytoalexins (in the similar scenario—comparative analyses of naïve and primed sorghum responses to the hemibiotrophic infection) would provide more experimental and confirmatory evidence in regards to this observation.

**Figure 6:**
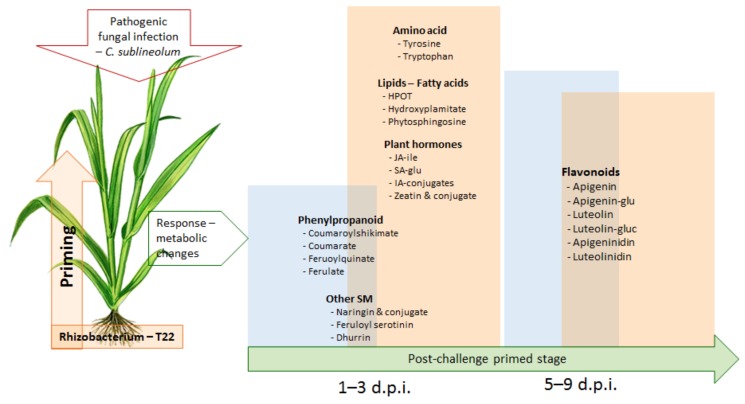
A contextual summary of results obtained in this study: A schematic diagram of comparative metabolic changes in PGPR primed and naïve sorghum plants during a post-challenge stage with the hemibiotrophic fungal pathogen, *C. sublineolum*. Following the rhizobacterium (*P. alvei*) application (24 h), sorghum plants were inoculated with *C. sublineolum* (blue = naïve plants and orange = primed plants). The primed plants showed a strong resistance to the fungal infection (symptomatology results), which was metabolically characterised by increased levels of aromatic amino acids, lipid components and phytohormones in the early phase of the post-challenge primed period: 1–3 d.p.i. Other metabolic changes observed during the post-challenge primed stage spanned different metabolic pathways particularly those involved in phenylpropanoid—and flavonoid synthesis.

In our previous report on the defence-related metabolic reprogramming in both naïve and *P. alvei*-primed *S. bicolor* plants in response to *Fusarium pseudograminearum* inoculation, the activation of both the early phenylpropanoid and flavonoid metabolic pathways as part of the induced systemic resistance response was observed [15]. This metabolic reconfiguration spans several metabolite classes including amino acids, phytohormones, lipids and phenolics. Although these classes of metabolites are similar to the ones identified in this study, there were some differences in timing, specific metabolites and levels of these metabolites. For instance, in response to *F. pseudograminearum*, discriminatory changes in amino acids were observed in lysine, histidine conjugates and glutamate [15]; whereas in response to *C. sublineolum*, alterations were observed mostly in tyrosine and tryptophan (Figure 4). Furthermore, indications of hormonal reprogramming involved gibberellins and hydroxyabscisic acid in regards to *F. pseudograminearum* infection [15], were not observed in this study. Pathogen-related metabolic changes were also observed in the phenolics and lipids involved in (naïve/primed) sorghum responses to the fungal infection [15] (Table 1). These differences suggest pathogen-specific spatial and temporal regulation of the sorghum defensome to the fungal infection. Thus, the emerging picture from the results of this study evidently demonstrates that the priming of sorghum by *P. alvei,* strain T22, pre-conditioned the plants to rapidly launch dynamic defence responses which span a range of primary and secondary metabolic pathways (Figure 6). Following the fungal infection (secondary challenge), the primed plant showed enhanced and quicker reprogramming of primary metabolite pools, modulation of resistance-related components of the lipidome, and a web of crosstalk between phytohormone pathways, for immediate strong reactions (as early as 1–3 d.p.i) to block the fungal invasion and prevent further proliferation. This was indicated by: (i) higher levels of amino acids, (Figure 4A and Figure 6) and their downstream derivatives (Table 1); (ii) higher levels of zeatin (and its glycosylated derivative) as well as the increased levels of JA- and SA-related compounds (Figure 4B, Figure 6 and Table 1); and (iii) the elevated levels of the lipid components in primed plants (vs. naïve plants), including hydroxy fatty acids and phytosphingosine (Figure 5A, Figure 6 and Table 1).

Moreover, the defence responses of both primed and naïve plants against the fungal infection were characterised by altered phenylpropanoid and flavonoid pathways, involving the *de novo* biosynthesis of the 3-deoxyanthocyanidin phytoalexins (luteolinidin and apigeninidin), luteolin and apigeninidin and some of the associated conjugates (Figure 5B, Figure 6 and Table 1). These observations from this study (depicted in Figure 6) correlate and contribute to the ongoing efforts in uncovering the underlying molecular mechanisms involved in priming events, with a focus on the post-challenge primed stage. As pointed out in the introduction, various studies have reported evidence that different priming stimuli induce metabolic reprogramming characterised by modulation in primary metabolism (during the priming phase): alterations in tricarboxylic acids fluxes and changes in amino acid and sugar biosynthesis, as well as potentiation of secondary metabolite biosynthesis [12,31,77]. This re-organised metabolism would serve to pre-condition the sorghum plants to launch a rapid and more effective defence response upon pathogen (secondary) challenge. Some of the metabolic changes observed during the post-challenge primed state (upon interaction with a pathogen) include such changes in phenolic metabolites and production of phytoalexins of various chemical classes [6,12].

## 3. Materials and Methods

### 3.1. Preparation of Sorghum Seedlings and Colletotrichum sublineolum Spore Suspensions

Sorghum [*Sorghum bicolor* (L.) Moench] seeds of a South African cultivar, NS 5655 (referred to as sweet, abbreviated herein as SWT) (Agricol, Pretoria, South Africa) was used. The cultivar is a grain sorghum hybrid of the malting class, and classified as GM (no condensed tannins, no dark testa). NS 5655 has a rating of ‘3’ (on a 1–9 scale with 1 being the most resistant) in displaying resistance against head smut, leaf disease and root rot (Capstone Seeds, Howick, South Africa). The cultivar is recognised by the Registrar of Plant Improvement, Department of Agriculture, Forestry and Fisheries, South Africa. Surface sterilisation of the seeds, germination and cultivation were as previously described [14]. The experimental plan was designed to monitor and compare the responses of primed and non-primed sorghum seedlings to *C. sublineolum* infection over time points of 1, 3, 5, 7 and 9-days post-infection (d.p.i.). The seedlings were planted in replicas of at least 10 plants per time point. Plants were grown under the same environmental conditions to minimise biological variability due to factors not related to the priming and fungal infection. The experimental design included three independent biological replicates.

An isolate of *C. sublineolum* (PPRI 7183) pathogenic on sorghum, was obtained from the National Collection of Fungi, Plant Protection Institute, Agricultural Research Council, Pretoria, South Africa. The fungus was grown and maintained on potato dextrose agar (PDA) and growth of the fungus for spore production was on 20% aqueous V8 vegetable juice media (pH 3.9) [14]. Harvested spores were diluted and the concentration was determined using a haemocytometer and light microscope at 400× magnification, and adjusted to 10^6^ spores mL^−1^.

### 3.2. Plant Growth Promoting Rhizobacteria Preparation and Inoculation of the Sorghum Seedlings

A PGPR, *Paenibacillus alvei* (strain T22), (obtained from the collection of Prof. N. Labuschagne, Department of Plant and Soil Science, University of Pretoria, South Africa) was tested for possible priming effects on the sorghum plants responding to *C. sublineolum* infection. This PGPR has previously been reported to successfully colonise tomato and wheat roots, and enhanced the growth of these plants [18]. The production of siderophores, indole-acetic acid and related compounds, and phosphate solubilisation were some of the elucidated mechanisms by which this bacterial isolate enhanced plant growth [18,78]. Pure bacterial cultures were prepared on Nutrient agar and used to inoculate sterile Nutrient broth medium (Biolab, Merck, Johannesburg, South Africa). The cultures were incubated for 48 h on a rotary shaker at 25 °C and 150 rpm. After the incubation, the PGPR concentration was determined and adjusted to 10^8^ cfu mL^−1^. The PGPR suspensions were applied to the vermiculite surrounding the plant roots at the 4-leaf growth stage. Control plants did not receive the PGPR treatment.

### 3.3. Secondary Challenge: Inoculation of Sorghum Seedlings with C. Sublineolum

To investigate the metabolomic reprogramming that describes the post-challenge primed state, the design of this study focussed on the simultaneous investigation of different conditions (naïve and primed sorghum plants, both responding to a fungal infection) in comparison to a non-treated control group. Following the 24 h PGPR application, the leaves of the PGPR-primed and naïve sorghum plants were treated by spraying with the fungal spore suspension (10^6^ spores mL^−1^, prepared as described [14]) until run-off, using a hand sprayer. Following inoculation, treated plants were incubated for 24 h at 30 °C in an incubator to provide 100% relative humidity. Subsequent to the 24 h incubation period, the plants were returned to the original initial conditions: with cycles of 12 h fluorescent light (≈85 µmol m^−2^ s^−1^) and 12 h darkness, and the temperature kept at 22–27 °C. Post-treatment harvesting was performed at 1, 3, 5, 7 and 9 d.p.i. and 1, 5, 9 d.p.i. for the non-treated plants (negative controls) [14]. Leaves were frozen with liquid Nitrogen for quenching of metabolic activity and stored at −80 °C until metabolite extraction.

### 3.4. Metabolite Extraction and Analyses by Ultrahigh Performance Liquid Chromatography-High Definition-Mass Spectrometry (UHPLC-HD-MS)

Metabolites were extracted from the PGPR-treated and naïve plants using 80% cold aqueous-methanol, in a ratio of 1:15 (*w*/*v*), at 4 °C. Homogenisation, concentration and reconstitution were as previously described [14]. Reconstituted samples were filtered through 0.22 µm nylon syringe filters and kept at −20 °C until analysed. The methanol used was LC-grade (Romil Pure Chemistry, Cambridge, UK) and ultrapure water. The quality control (QC) samples were pooled samples prepared from mixed aliquots of equal volume from all samples.

Analytical separation of the extracts was performed on a Waters Acquity HSS T3 C18 chromatography column (150 mm × 2.1 mm ×1.8 µm) thermostatted at 60 °C, on a Waters Acquity UHPLC coupled in tandem to a Waters SYNAPT G1 Q-TOF mass spectrometer (Waters Corporation Milford, CT, USA). Analyses were conducted with a binary solvent system consisting of 0.1% aqueous formic acid (Sigma-Aldrich, Munich, Germany) (solvent A) and 0.1% formic acid in acetonitrile (Romil Pure Chemistry, Cambridge, UK) (solvent B) at a flow rate of 0.4 mL min^−1^. Conditions for the gradient elution were: 0–1 min 2% B, 14 min 70% B, 15–17 min 95% B, 18 min 2% B, with the column allowed to calibrate for 2 min before the next injection. The total chromatographic run time was 20 min and the injection volume was 2 µL. The LC eluents were detected and further analysed by mass spectrometry on a SYNAPT G1 Q-TOF MS system in V-optics operated in both ESI(+/−) modes. Leucine encephalin (50 pg mL^−1^), [M + H]^+^ = 55.2766 and [M − H]^−^ = 554.2615, served as a reference calibrant, producing an average intensity of 350 counts scan^−1^ in centroid mode, and giving typical mass accuracies between 1–3 mDa. Instrumental settings and conditions of the mass spectrometer were as previously described [14]. The data were acquired with different collision energies (MS^E^) 0–30 eV to generate increased fragmentation of the molecular ions so as to obtain as much fragment-based structural information as possible of the detected compounds [79].

The software used to control the hyphenated system and perform all data manipulation was MassLynx-XS^TM^ 4.1 (Waters Corporation Milford, USA). To account for any analytical variability, each sample was analysed in triplicate. The QC samples were used to condition the LC-MS system, and to assess the reliability and reproducibility of the analysis: 6 QC runs at the beginning and end of the batch and 6 QC injections every 10 injections. Randomisation was applied in sample acquisition order. Blanks were injected to monitor background noise or any solvent-related contamination.

### 3.5. Data Analysis: Data Set Matrix Creation and Chemometric Analyses

The MarkerLynx-XS^TM^ application manager of the MassLynx-XS^TM^ 4.1 software (Waters Corporation, Manchester, UK) was used for data pre-processing (matrix creation), producing a matrix with rows representing the individual mass spectra, and columns representing retention time (Rt)-*m*/*z* variable pairs, with integrated and normalised peak areas. MarkerLynx software parameters were set to process the 1–15 min Rt range of the chromatograms and *m*/*z* domain of mass range 100–1000 Da. The Rts were allowed to differ by ±0.2 min and the *m*/*z* values by ±0.05 Da. The mass tolerance used was 0.01 Da, and the intensity threshold was 100 counts. Only data matrices that had noise levels less than 50% (MarkerLynx cut-off) were retained for downstream chemometric and statistical analyses. The MarkerLynx-generated data matrices were imported into SIMCA software, version 14 (Umetrics, Umeå, Sweden) for chemometric analyses: employing mostly two unsupervised methods, PCA and HCA, and a supervised modelling, OPLS-DA. For variable selection, OPLS-DA-generated loadings S-plots and SUS-plots were evaluated. A nonlinear iterative partial least squares algorithm (within the SIMCA software) [80] was used to manage the missing values, with a correction factor of 3.0 and a default threshold of 50%. A seven-fold cross-validation (CV) method [81] was applied as a tuning procedure in computing the chemometric models, and only the components positively contributing to increase the prediction ability of the model (*R1* significant components) were considered. Moreover, model validations were rigorously and consistently applied, and only statistically (chemometrically) satisfactory models were examined and used in data mining.

### 3.6. Metabolite Annotation: Putative Identification of Chemometrically Selected Metabolites

The data matrices from MarkerLynx-based data processing were exported to the Taverna workbench for PUTMEDID_LCMS Metabolite ID Workflows [82,83]. The Taverna workflows allow for integrated, automated and high-throughput annotation and putative metabolite identification from LC-ESI-MS metabolomic data. The Taverna Metabolite ID procedure consists of three main workflows: (i) Pearson-based correlation analysis (*List_CorrData*), (ii) metabolic feature annotation (*annotate_Massmatch*)—allowing for grouping together ion peaks with similar features such as Rt, and annotating features with the type of *m*/*z* ion (molecular ion, isotope, adduct, others) believed to originate from the same compound. The elemental composition/molecular formula (MF) of each *m*/*z* ion is then automatically calculated; and (iii) metabolite annotation (*matchMF-MF*) of the calculated MF (from the output file from workflow ii) is automatically compared and matched to the MF from a pre-defined reference file of metabolites.

The following steps were performed for confidence in metabolite annotation: (i) the calculated molecular formula of a selected metabolite candidate was manually searched against databases and bioinformatics tools, mainly the Dictionary of Natural Products (DNP), Chemspider, the Plant Metabolic Network—PlantCyc, Knapsack and the Kyoto Encyclopedia of Genes and Genomes (KEGG) [84] (ii) structural confirmation through inspection of fragmentation patterns by examining the MS^1^ and MS^E^ spectra of the selected metabolite candidate; and (iii) comparative assessment with/against annotation details of metabolites in sorghum, reported in literature, particularly in [83,84]. Metabolites were annotated to MSI level 2 as classified by the Metabolomics Standard Initiative [85].

## 4. Conclusions and Perspectives

The priming phenomenon temporally involves different stages; the priming stage, a post-challenge primed state and a transgenerational primed state. Detailed molecular mechanisms underlying each stage remain elusive. Thus, the main focus of this study was to characterise the underlying metabolic reprogramming related to the priming effects of a PGPR, the rhizobacterium *P. alvei* (T22), in sorghum plants responding to an infection with a hemibiotrophic fungal pathogen, *C. sublineolum*. By employing an LC-MS untargeted metabolomics approach of hydromethanolic-extracted metabolites, the study revealed strong defence-related metabolic reprogramming observed in primed plants compared to naïve plants as early as 1–3 d.p.i., pointing to the molecular preparedness of the primed plants to rapidly halt the invasion and establishment of *C. sublineolum*. Evaluation of SUS-analyses pointed out that the differential stronger defence responses against the fungal infection observed in *P. alvei* (T22)-primed sorghum plants were mostly characterised by increased levels of aromatic amino acids, phytohormones and defence-related components of the lipidome. Thus, the study showed that the PGPR-induced priming of the sorghum plants potentiated the latter to speedily launch defence mechanisms. These involved reprogramming of aspects of primary metabolism, reconfiguration of the cellular lipidome and an intricate web of crosstalk between different phytohormone pathways for immediate strong reactions to halt the fungal invasion. Furthermore, other defence responses in both naïve and primed plants involved an altered metabolism that spans a number of metabolic routes, with the centrality of both phenylpropanoid and flavonoid pathways. These changes included a complex mobilisation of phenolic compounds and *de novo* biosynthesis of 3-deoxyanthocyanidin phytoalexins (apigeninidin, luteolinidin), apigenin, luteolin and some of the associated conjugates.

## Figures and Tables

**Table 1:** Summary of annotated (MSI-level 2) metabolites that contributed to the discriminating variability in the altered metabolomes as described by chemometric models. These discriminating, putatively identified metabolites were identified based on OPLS-DA S-plots and SUS-plots, with a rigorous statistical validation. These reported metabolites had *p*-values < 0.05 and VIP scores > 1.0. Fragmentation data and applicable references are reported in Table S1.

	Metabolites	*m*/*z*	Rt (min)	Adduct	ESI Mode	Molecular Formula	Biochemical Classification	Post-Challenge Period (Primed/Naïve)			
								1–3 d.p.i.		5–9 d.p.i.	
								*p*-Value	Fold Change	*p*-Value	Fold Change
1	L-Tyrosine	182.0819	1.25	H	pos	C_9_H_11_NO_3_	Amino acid	7.97 × 10^−7^	88.41	1.68 × 10^−10^	0.52
2	5-Hydroxytryptophan	236.1036	2.65	NH3	neg	C_11_H_12_N_2_O_3_	Amino acid	1.33 × 10^−10^	89.28	1.34 × 10^−11^	1.99
3	L-Tryptophan	205.0978	3.02	H	pos	C_11_H_12_N_2_O_2_	Amino acid	5.91 × 10^−11^	2.85	2.48 × 10^−10^	0.57
4	Dhurrin	329.1335	4.02	NH_3_	pos	C_14_H_17_NO_7_	Cyanogenic glucoside	7.68 × 10^−27^	40.50	4.91 × 10^−8^	24.80
5	Naringin chalcone	627.1912	2.52	HCOOH	neg	C_27_H_34_O_14_	Flavonoid	1.50 × 10^−7^	6.13	3.34 × 10^−10^	1.30
6	Naringin	625.1761	3.46	HCOOH	neg	C_27_H_32_O_14_	Flavonoid	5.17 × 10^−7^	5.46	3.60 × 10^−10^	2.08
7	Peptahydroxychalcone 4′-*O*-glucoside	449.1067	4.73	H	neg	C_21_H_22_O_11_	Flavonoid	1.88 × 10^−15^	2.29	8.14 × 10^−10^	1.60
8	Hesperidin	609.1809	5.00	H	neg	C_28_H_34_O_15_	Flavonoid	3.43 × 10^−11^	2.22	2.36 × 10^−9^	0.74
9	Apigenin 7-*O*-[beta-d-apiosyl-(1->2)-beta-d-glucoside]	563.1390	5.05	H	neg	C_26_H_28_O_14_	Flavonoid	4.51 × 10^−4^	1.09	1.03 × 10^−14^	4.84
10	Kaempferol 3-*O*-rhamnoside-7-*O*-glucoside	593.1501	5.68	H	neg	C_27_H_30_O_15_	Flavonoid	2.75 × 10^−4^	3.24	1.53 × 10^−14^	0.36
11	Cyanidin 3-*O*-rhamnosylglucoside	595.1657	5.75	H	pos	C_27_H_30_O_15_	Flavonoid	1.74 × 10^−14^	0.34	4.06 × 10^−19^	0.22
12	Kaempferol-3-glucoside	447.0921	5.88	H	neg	C_21_H_20_O_11_	Flavonoid	1.97 × 10^−4^	3.22	2.84 × 10^−14^	4.94
13	Quercitrin	449.1080	5.92	H	pos	C_21_H_20_O_11_	Flavonoid	2.08 × 10^−4^	1.14	2.28 × 10^−14^	0.22
14	Apigenin	271.1544	6.02	H	pos	C_15_H_10_O_5_	Flavonoid	8.82 × 10^−4^	0.30	2.01 × 10^−7^	0.36
15	Apigeninidin	255.1533	6.10	H	pos	C_15_H_11_O_4_	Flavonoid	6.84 × 10^−24^	0.31	1.10 × 10^−32^	0.77
16	Luteolin 7-*O*-beta-d-glucoside	447.0921	6.19	H	neg	C_21_H_20_O_11_	Flavonoid	8.27 × 10^−5^	1.12	2.95 × 10^−12^	0.27
17	Apigenin 7-*O*-neohesperidoside	579.1709	6.27	H	pos	C_27_H_30_O_14_	Flavonoid	1.86 × 10^−11^	1.00	3.06 × 10^−31^	0.20
18	Luteolin	287.0536	6.30	H	pos	C_15_H_10_O_6_	Flavonoid	4.25 × 10^−23^	0.36	0.000	0.51
19	1,2-bis-*O*-Sinapoyl-beta-d-glucoside	591.1705	6.35	H	neg	C_28_H_32_O_14_	Flavonoid	1.28 × 10^−29^	3.17	5.02 × 10^−14^	10.39
20	7-*O*-Methylvitexin 2′′-*O*-beta-l-rhamnoside	615.1680	6.39	Na	pos	C_28_H_32_O_14_	Flavonoid	4.35 × 10^−13^	0.31	1.09 × 10^−25^	0.07
21	Isovitexin 2′′-*O*-beta-d-glucoside	593.1501	6.68	H	neg	C_27_H_30_O_15_	Flavonoid	7.22 × 10^−3^	1.96	1.08 × 10^−14^	3.65
22	Luteolinidin	271.0616	6.87	H	pos	C_15_H_11_O_5_	Flavonoid	8.23 × 10^−3^	0.36	7.68 × 10^−27^	0.68
23	12,13-Epoxy-9-hydroxy-10-octadecenoate	395.2040	9.26	HCOONa	neg	C_18_H_32_O_5_	Lipid	1.52 × 10^−17^	50.98	5.07 × 10^−4^	39.73
24	Phytosphingosine	318.3009	10.52	H	pos	C_18_H_39_NO_3_	Lipid	1.29 × 10^−29^	267.28	0.000	27.50
25	16-Hydroxypalmitate	290.2700	10.58	NH3	pos	C_16_H_32_O_3_	Lipid	7.30 × 10^−12^	217.16	1.42 × 10^−19^	24.76
26	(9Z)-(13S)-12,13-Epoxyoctadeca-9,11-dienoic acid	363.2137	11.44	HCOONa	pos	C_18_H_30_O_3_	Lipid	1.96 × 10^−14^	139.22	8.66 × 10^−11^	35.53
27	13(S)-hydroxyperoxy-octadecatrienoic acid	309.2071	11.79	H	neg	C_18_H_30_O_4_	Lipid	1.02 × 10^−10^	40.98	1.84 × 10^−5^	28.80
28	25-Hydroxy-24-epi-brassinolide	519.3267	13.34	Na	pos	C_28_H_48_O_7_	Lipid	9.77 × 10^−14^	35.74	1.47 × 10^−6^	28.16
29	Oleanolate 3-beta-d-glucuronoside-28-glucoside	795.4497	15.36	H	pos	C_42_H_66_O_14_	Lipid	2.32 × 10^−22^	31.31	1.94 × 10^−14^	19.49
30	Oleanoic acid 3-*O*-glucuronide	655.3820	15.40	Na	pos	C_36_H_56_O_9_	Lipid	0.000	36.35	1.58 × 10^−37^	18.05
31	Caffeoylquinate	377.0846	3.83	Na	pos	C_16_H_18_O_9_	Phenylpropanoid	3.64 × 10^−12^	12.59	1.35 × 10^−28^	9.05
32	p-Coumaroyl quinic acid	427.0621	1.03	NaHCOONa	neg	C_16_H_18_O_8_	Phenylpropanoid	4.06 × 10^−6^	13.20	1.94 × 10^−27^	6.75
33	Feruloyltyramine	331.1650	2.01	NH_3_	pos	C_18_H_19_NO_4_	Phenylpropanoid	5.02 × 10^−14^	10.39	2.38 × 10^−20^	11.63
34	4-Coumaroylshikimate	319.1062	3.16	H	neg	C_16_H_16_O_7_	Phenylpropanoid	2.46 × 10^−7^	12.33	3.41 × 10^−19^	0.58
35	2-Coumarate	165.0554	3.25	H	pos	C_9_H_8_O_3_	Phenylpropanoid	2.16 × 10^−17^	8.78	2.20 × 10^−20^	0.58
36	1-*O*-Sinapoyl-beta-d-glucose	387.1279	3.56	H	pos	C_17_H_22_O_10_	Phenylpropanoid	3.23 × 10^−9^	14.50	6.72 × 10^−20^	0.59
37	4-*O*-beta-d-Glucosyl-4-hydroxycinnamate	395.0947	4.09	HCOONa	pos	C_15_H_18_O_8_	Phenylpropanoid	7.40 × 10^−5^	14.12	9.53 × 10^−20^	1.60
38	Ferulate	209.0448	4.58	H	neg	C_10_H_10_O_5_	Phenylpropanoid	9.42 × 10^−6^	12.13	5.37 × 10^−25^	0.47
39	*O*-Feruloylquinate	367.1017	4.88	H	neg	C_17_H_20_O_9_	Phenylpropanoid	2.38 × 10^−20^	11.63	9.62 × 10^−23^	0.49
40	Coniferyl acetate	291.0844	1.09	HCOONa	pos	C_12_H_14_O_4_	Phenylpropanoid	9.28 × 10^−7^	12.52	9.86 × 10^−19^	2.41
41	Zeatin	220.1192	2.38	H	pos	C_10_H_13_N_5_O	Phytohormone	0.000	17.09	3.19 × 10^−21^	14.17
42	Salicylate-glucoside	299.0758	1.79	H	neg	C_13_H_16_O_8_	Phytohormone	1.43 × 10^−4^	9.19	2.01 × 10^−17^	1.66
43	6-Hydroxy-indole-3-acetyl-phenylalanine	405.1077	2.76	HCOONa	neg	C_19_H_18_N_2_O_4_	Phytohormone	1.38 × 10^−4^	9.36	1.51 × 10^−17^	2.75
44	6-Hydroxy-indole-3-acetyl-valine	335.0962	2.82	Na_Na	pos	C_15_H_18_N_2_O_4_	Phytohormone	3.08 × 10^−6^	10.71	2.83 × 10^−16^	4.88
45	(-)-Jasmonoyl-l-isoleucine	406.1626	4.33	HCOOK	neg	C_18_H_29_NO_4_	Phytohormone	6.08 × 10^−9^	8.21	2.16 × 10^−17^	8.78
46	12-Hydroxyjasmonic acid 12-*O*-beta-d-glucoside	429.1514	5.59	Na_Na	neg	C_19_H_30_O_8_	Phytohormone	1.57 × 10^−6^	10.73	1.14 × 10^−16^	2.76
47	*trans*-Zeatin-7-beta-d-glucoside	399.1990	8.14	NH_3_	pos	C_16_H_23_N_5_O_6_	Phytohormone	6.82 × 10^−6^	9.53	2.98 × 10^−16^	0.46
48	Riboflavin	419.0969	5.80	Na_Na	neg	C_17_H_20_N_4_O_6_	Riboflavin	1.42 × 10^−19^	24.76	2.84 × 10^−7^	18.67
49	Feruloylserotonin	351.1333	11.66	H	neg	C_20_H_20_N_2_O_4_	Trp pathway	1.44 × 10^−3^	23.73	1.09 × 10^−25^	15.32

## Data Availability

The study design information, LC-MS raw data, analyses and data processing information, and the meta-data were deposited to the EMBL-EBI metabolomics repository—MetaboLights50, with the identifier MTBLS1176 (https://www.ebi.ac.uk/metabolights/MTBLS1176).

## Supplementary Materials

The following are available online at https://www.mdpi.com/2218-1989/9/10/194/s1, Figure S1: Evaluation of disease symptoms in *Colletotrichum sublineolum* infected sorghum plants; Figure S2: Representative BPI MS chromatograms of ESI(+) data (3 d.p.i.); Figure S3: Unsupervised chemometric modelling of ESI(-) data; Figure S4: OPLS-DA modelling and variable/feature selection. Table S1: Annotated (MSI-level 2) metabolites reported in Table 1, with fragmentation information.
