# Supplementary material for: Unravelling the Metabolic Reconfiguration of the Post-Challenge Primed State in Sorghum bicolor Responding to Colletotrichum sublineolum Infection
Source: Metabolites. 2019 Sep 20;9(10):194. doi: 10.3390/metabo9100194 (PMC6835684; doi:10.3390/metabo9100194)
Supplement: Supplementary file 1 [file metabolites-09-00194-s001.pdf]

Supplementary Materials

Unravelling the metabolic reconfiguration of the post-challenge primed state in *Sorghum bicolor* responding to *Colletotrichum sublineolum* infection

Fidele Tugizimana [1], Paul A. Steenkamp [1], Lizelle A. Piater [1], Nico Labuschagne [2] and Ian A. Dubery [1,*]

[1] Research Centre for Plant Metabolomics, Department of Biochemistry, University of Johannesburg, Auckland Park, South Africa

[2] Department of Plant and Soil Science, University of Pretoria, Pretoria, South Africa

* Correspondence: idubery@uj.ac.za; Tel.: + 27-011-559-2401.

Figure S1. Evaluation of disease symptoms in *Colletotrichum sublineolum* infected sorghum plants.

Figure S2. Representative MS chromatograms of ESI(+) data (3 d.p.i.).

Figure S3. Unsupervised chemometric modelling of ESI(-) data.

Figure S4. OPLS-DA modelling and variable/feature selection.

Table S1. Annotated (MSI-level 2) metabolites reported in Table 1, with fragmentation information (refs. [1-11]).

References [1] – [14].

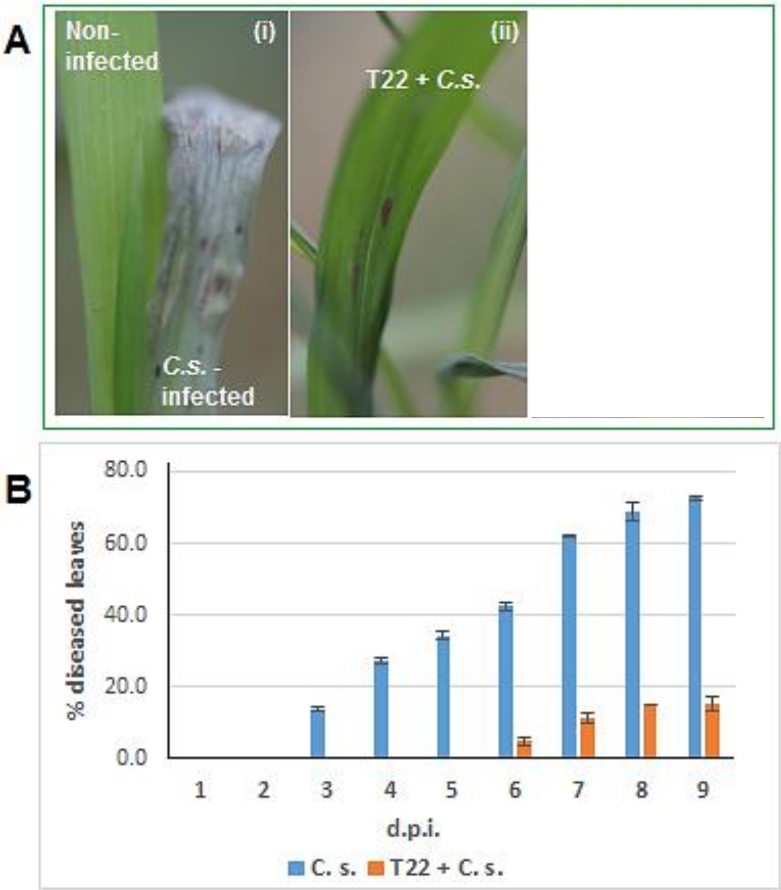

Figure S1. Evaluation of disease symptoms in *Colletotrichum sublineolum* infected sorghum plants. (**A**): Symptoms observed on sorghum leaves following the fungal challenge. **(i)** The sorghum plants that were not inoculated with a bacterial suspension show anthracnose symptoms that were severe at late stage of the disease development (9 d.p.i.). **(ii)** The sorghum plants inoculated with the *Paenibacillus alvei* (T22) bacterial strain, then challenged with *C. sublineolum*. The symptoms developed very late (from 6 d.p.i.) and were very few even at 9 d.p.i.. Chlorosis and wilting of the leaf could be observed. Purple spots indicate the accumulation of 3-deoxyanthocyanidins. (**B**): The rating of the symptom development over time (1 – 9 d.p.i.). The disease symptoms were scored by measuring the percentage of infected leaves relative to the total number of analysed leaves. The values are the means of the percentage of diseased leaves per plant ± SD.

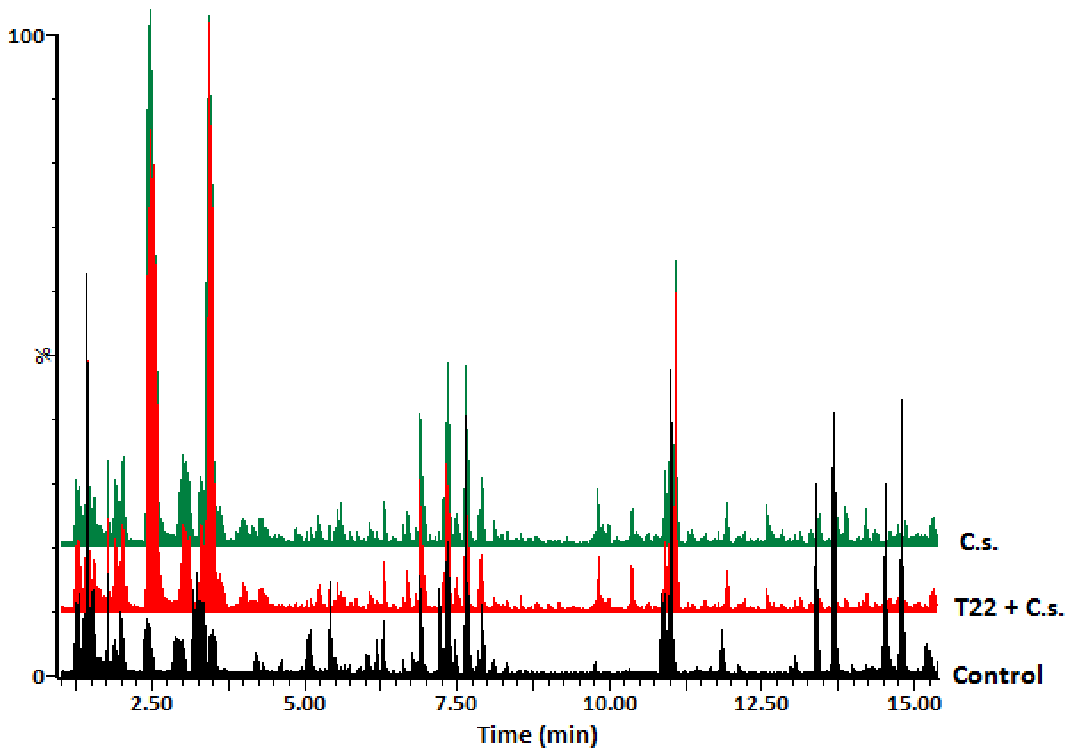

Figure S2. Representative MS chromatograms of ESI(+) data (3 d.p.i.). Base peak intensity (BPI) mass chromatograms displaying comparative chromatographic differences in different conditions: (**i**) samples from non-treated plants (Control, NT), (**ii**) samples from *Paenibacillus alvei* (T22)-primed and *C. sublineolum* (*C.s.*)-challenged plants and (**iii**) samples from *C.s.*-infected plants. Visual inspection of the chromatograms evidently shows differential peak populations, for instance in the 4–12 min chromatographic region.

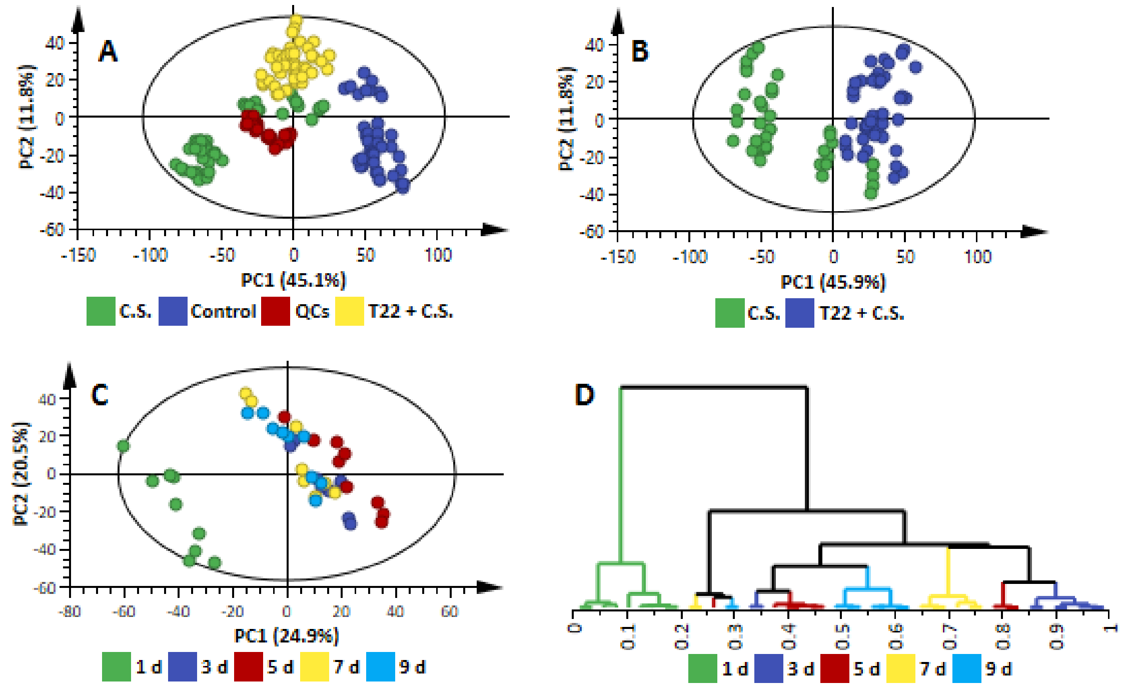

Figure S3. Unsupervised chemometric modelling of ESI(-) data:

(**A**): A PCA scores scatter plot (first two components) of a 12-component model, explaining 85.1% of the variation in *Pareto*-scaled data X (of all the samples, including the QC samples), and predicting 74.4% variation, according to cross-validation; and coloured according to the treatment: non-treated (control - blue), fungal treated plants (*C.s.* - green), *Paenibacillus alvei* (T22)-primed and challenged with *C. sublineolum* plants (T22 + *C.s.* - yellow). The scores plot shows treatment-related grouping, and the QC samples (brown) clustered together, indicating the reliability and good quality of the acquired data.

(**B**): Explorative analyses of the ESI(-) data of samples from fungal treated plants (*C.s.* - green) and T22-primed and challenged with *C. sublineolum* plants (blue). The PCA scores plot of an 8-component model (R^2 = 0.803 and Q^2 = 0.677) show treatment-related sample grouping, pointing to differential metabolic changes in the two treatment groups.

(**C**): A PCA scores plot of a 6-component model, explaining 70.8% of the variation in *Pareto*-scaled data X (of T22 + *C.s.* – primed and infected samples), with a predictive ability of 69.7%, according to cross-validation. The scores plot indicates time-related grouping, pointing to time-dependent metabolic changes in the response of T22-primed sorghum plants responding to the fungal infection.

(**D**): HCA dendrogram corresponding to (**C**), allowing the identification of natural clustering in multivariate metabolite space: treatment-related grouping.

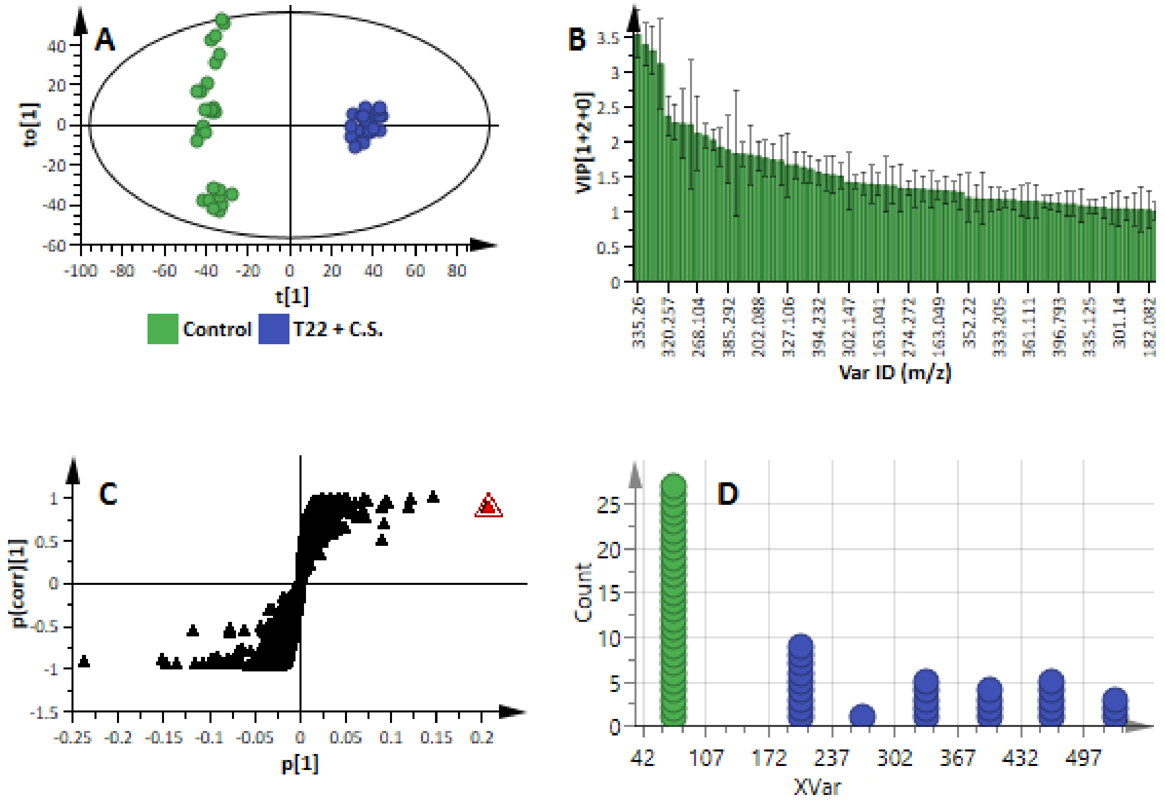

Figure S4. OPLS-DA modelling and variable/feature selection.

(**A**): A typical OPLS-DA scores scatter plot of the OPLS-DA model of ESI(-) data, separating 'control plants *vs*. challenged primed-plants' at 5-9 d.p.i. (1 + 2 + 0 components, $R^2X = 0.658$, $Q^2 = 0.978$, CV-ANOVA *p*-value = 0.00) (**Figure 3**). In the scores space, the two groups are clearly separated.

(**B**): A typical VIP plot (from the same OPLS-DA model as in **Figure 3**). Such a plot allows the identification / selection of variables (features) with high importance in driving the separation of the two groups in the binary model.

(**C**): A typical loadings S-plot used to select discriminating variables (features). The variables with high covariance and high correlation (those found in both extreme ends of the S-plot) are of interest – *e.g*. the red feature.

(**D**): A typical dot plot (of the red feature in S-plot) which allows the assessment of the discriminability of the selected variable. As displayed by the dot plot, the selected variable is a perfect discriminating feature, as no overlap can be seen in the separated groups.

Table S1. Annotated (MSI-level 2) metabolites reported in **Table 1**, with fragmentation information (refs. [1-14]).

	Metabolites	Rt (min)	*m/z*	Fragmentation	Adduct	mode	MF	Class
**1**	L-Tyrosine	1.25	182.0819	136	H	pos	$C_9H_{11}NO_3$	Amino acid
**2**	5-Hydroxytryptophan	2.65	236.1036	188	NH3	neg	$C_{11}H_{12}N_2O_3$	Amino acid
**3**	L-Tryptophan	3.02	205.0978	188	H	pos	$C_{11}H_{12}N_2O_2$	Amino acid
**4**	Dhurrin	4.02	329.1335	185, 307	NH_3	pos	$C_{14}H_{17}NO_7$	Cyanogenic glucoside
**5**	Naringin chalcone	2.52	627.1912	339, 315, 273	HCOOH	neg	$C_{27}H_{34}O_{14}$	Flavonoid
**6**	Naringin	3.46	625.1761	271, 151	HCOOH	neg	$C_{27}H_{32}O_{14}$	Flavonoid
**7**	Peptahydroxychalcone 4'-O-glucoside	4.73	449.1067	207, 287	H	neg	$C_{21}H_{22}O_{11}$	Flavonoid
**8**	Hesperidin	5	609.1809	343, 301, 179, 151	H	neg	$C_{28}H_{34}O_{15}$	Flavonoid
**9**	Apigenin 7-O-[beta-D-apiosyl-(1->2)-beta-D-glucoside]	5.05	563.139	269, 225, 197	H	neg	$C_{26}H_{28}O_{14}$	Flavonoid
**10**	Kaempferol 3-O-rhamnoside-7-O-glucoside	5.68	593.1501	447, 285	H	neg	$C_{27}H_{30}O_{15}$	Flavonoid
**11**	Cyanidin 3-O-rhamnosylglucoside	5.75	595.1657	449, 300, 287	H	pos	$C_{27}H_{30}O_{15}$	Flavonoid
**12**	Kaempferol-3-glucoside	5.88	447.0921	285	H	neg	$C_{21}H_{20}O_{11}$	Flavonoid
**13**	Quecertin	5.92	301.0367	273, 193, 179, 151	H	neg	$C_{15}H_{10}O_7$	Flavonoid
**14**	Apigenin	6.02	271.1544	243, 227, 203, 153, 109	H	pos	$C_{15}H_{10}O_5$	Flavonoid
**15**	Apigeninidin	6.1	255.1533	227, 181, 171, 157, 115	H	pos	$C_{15}H_{11}O_4$	Flavonoid
**16**	Luteolin 7-O-beta-D-glucoside	6.19	447.0921	357, 287, 153, 135, 117	H	neg	$C_{21}H_{20}O_{11}$	Flavonoid
**17**	Apigenin 7-O-neohesperidoside	6.27	579.1709	227, 153	H	pos	$C_{27}H_{30}O_{14}$	Flavonoid
**18**	Luteolin	6.3	287.0536	259, 243, 201, 177	H	pos	$C_{15}H_{10}O_6$	Flavonoid
**19**	1,2-Bis-O-sinapoyl-beta-D-glucoside	6.35	591.1705	367, 206	H	neg	$C_{28}H_{32}O_{14}$	Flavonoid
**20**	7-O-Methylvitexin 2''-O-beta-L-rhamnoside	6.39	615.168	225, 187, 115	Na	pos	$C_{28}H_{32}O_{14}$	Flavonoid
**21**	Isovitexin 2''-O-beta-D-glucoside	6.68	593.1501	473, 447	H	neg	$C_{27}H_{30}O_{15}$	Flavonoid
**22**	Luteolinidin	6.87	271.0616	225, 197, 187, 115	H	pos	$C_{15}H_{11}O_5$	Flavonoid
**23**	12,13-Epoxy-9-hydroxy-10-octadecenoate	9.26	395.204	197, 171	HCOONa	neg	$C_{18}H_{32}O_5$	Lipid
**24**	Phytosphingosine	10.52	318.3009	---	H	pos	$C_{18}H_{39}NO_3$	Lipid

**25**	16-Hydroxypalmitate	10.58	290.27	229, 173, 159, 145	NH3	pos	$C_{16}H_{32}O_3$	Lipid
**26**	(9Z)-(13S)-12,13-Epoxyoctadeca-9,11-dienoic acid	11.44	363.2137	---	HCOONa	pos	$C_{18}H_{30}O_3$	Lipid
**27**	13(S)-hydroxyperoxyoctadecatrienoic acid	11.79	309.2071	314, 135	H	neg	$C_{18}H_{30}O_4$	Lipid
**28**	25-Hydroxy-24-epi-brassinolide	13.34	519.3267	---	Na	pos	$C_{28}H_{48}O_7$	Lipid
**29**	Oleanolate 3-beta-D-glucuronoside-28-glucoside	15.36	795.4497	---	H	pos	$C_{42}H_{66}O_{14}$	Lipid
**30**	Oleanoic acid 3-O-glucuronide	15.4	655.382	---	Na	pos	$C_{36}H_{56}O_9$	Lipid
**31**	Caffeoylquinate	3.83	377.0846	193, 181, 175, 121	Na	pos	$C_{16}H_{18}O_9$	Phenylpropanoid
**32**	p-Coumaroyl quinic acid	1.03	427.0621	337, 191, 173	NaHCOONa	neg	$C_{16}H_{18}O_8$	Phenylpropanoid
**33**	Feruloyltyramine	2.01	331.165	314, 192, 180, 137	NH_3	pos	$C_{18}H_{19}NO_4$	Phenylpropanoid
**34**	4-Coumaroylshikimate	3.16	319.1062	301, 283, 163, 119	H	neg	$C_{16}H_{16}O_7$	Phenylpropanoid
**35**	2-Coumarate	3.25	165.0554	119	H	pos	$C_9H_8O_3$	Phenylpropanoid
**36**	1-O-Sinapoyl-beta-D-glucose	3.56	387.1279	225, 207, 181	H	pos	$C_{17}H_{22}O_{10}$	Phenylpropanoid
**37**	4-O-beta-D-Glucosyl-4-hydroxycinnamate	4.09	395.0947	217, 193, 175	HCOONa	pos	$C_{15}H_{18}O_8$	Phenylpropanoid
**38**	Ferulate	4.58	209.0448	194, 178, 161, 134	H	neg	$C_{10}H_{10}O_5$	Phenylpropanoid
**39**	O-Feruloylquinate	4.88	367.1017	193,173,134	H	neg	$C_{17}H_{20}O_9$	Phenylpropanoid
**40**	Coniferyl acetate	1.09	291.0844	182	HCOONa	pos	$C_{12}H_{14}O_4$	Phenylpropanoid
**41**	Zeatin	2.38	220.1192	202, 136, 119	H	pos	$C_{10}H_{13}N_5O$	Phytohormone
**42**	Salicylate-glucoside	1.79	299.0758	136, 92	H	neg	$C_{13}H_{16}O_8$	Phytohormone
**43**	6-Hydroxy-indole-3-acetyl-phenylalanine	2.76	405.1077	120, 103, 93	HCOONa	neg	$C_{19}H_{18}N_2O_4$	Phytohormone
**44**	6-Hydroxy-indole-3-acetyl-valine	2.82	335.0962	173, 129	Na_Na	pos	$C_{15}H_{18}N_2O_4$	Phytohormone
**45**	(-)-Jasmonoyl-L-isoleucine	4.33	406.1626	338, 336, 301	HCOOK	neg	$C_{18}H_{29}NO_4$	Phytohormone
**46**	12-Hydroxyjasmonic acid 12-O-beta-D-glucoside	5.59	429.1514	370, 267	Na_Na	neg	$C_{19}H_{30}O_8$	Phytohormone
**47**	*trans*-Zeatin-7-beta-D-glucoside	8.14	399.199	202, 181, 136, 119	NH_3	pos	$C_{16}H_{23}N_5O_6$	Phytohormone
**48**	Riboflavin	5.8	419.0969	398, 381, 355, 224, 143	Na_Na	neg	$C_{17}H_{20}N_4O_6$	Riboflavin
**49**	Feruloylserotonin	11.66	351.1333	177, 149, 145	H	neg	$C_{20}H_{20}N_2O_4$	Trp pathway

References

1. Kang, J.; Price, W. E.; Ashton, J.; Tapsell, L. C.; Johnson, S. Identification and characterization of phenolic compounds in hydromethanolic extracts of sorghum wholegrains by LC-ESI-MSn. *Food Chem.* **2016**, *211*, 215–226, doi:10.1016/j.foodchem.2016.05.052.
2. van der Hooft, J. J. J.; Vervoort, J.; Bino, R. J.; Beekwilder, J.; de Vos, R. C. H. Polyphenol identification based on systematic and robust high-resolution accurate mass spectrometry fragmentation. *Anal. Chem.* **2011**, *83*, 409–416, doi:10.1021/ac102546x.
3. Jaiswal, R.; Müller, H.; Müller, A.; Karar, M. G. E.; Kuhnert, N. Identification and characterization of chlorogenic acids, chlorogenic acid glycosides and flavonoids from *Lonicera henryi* L. (Caprifoliaceae) leaves by LC–MSn. *Phytochemistry* **2014**, *108*, 252–263, doi:10.1016/j.phytochem.2014.08.023.
4. Moco, S.; Bino, R. J.; Vorst, O.; Verhoeven, H. A.; de Groot, J.; van Beek, T. A.; Vervoort, J.; de Vos, C. H. R. A liquid chromatography-mass spectrometry-based metabolome database for tomato. *Plant Physiol.* **2006**, *141*, 1205–1218, doi:10.1104/pp.106.078428.
5. Vallverdú-Queralt, A.; Jáuregui, O.; Medina-Remón, A.; Andrés-Lacueva, C.; Lamuela-Raventós, R. M. Improved characterization of tomato polyphenols using liquid chromatography/electrospray ionization linear ion trap quadrupole Orbitrap mass spectrometry and liquid chromatography/electrospray ionization tandem mass spectrometry. *Rapid Commun. Mass Spectrom.* **2010**, *24*, 2986–2992, doi:10.1002/rcm.4731.
6. Matsuda, F.; Yonekura-Sakakibara, K.; Niida, R.; Kuromori, T.; Shinozaki, K.; Saito, K. MS/MS spectral tag-based annotation of non-targeted profile of plant secondary metabolites. *Plant J.* **2009**, *57*, 555–77, doi:10.1111/j.1365-313X.2008.03705.x.
7. Nikolić, D.; Gödecke, T.; Chen, S. N.; White, J.; Lankin, D. C.; Pauli, G. F.; Van Breemen, R. B. Mass spectrometric dereplication of nitrogen-containing constituents of black cohosh (*Cimicifuga racemosa* L.). *Fitoterapia* **2012**, *83*, 441–460, doi:10.1016/j.fitote.2011.12.006.
8. Ratzinger, A.; Riediger, N.; von Tiedemann, A.; Karlovsky, P. Salicylic acid and salicylic acid glucoside in xylem sap of *Brassica napus* infected with *Verticillium longisporum*. *J. Plant Res.* **2009**, *122*, 571–579, doi:10.1007/s10265-009-0237-5.
9. Cho, K.; Kim, Y.; Wi, S. J.; Seo, J. B.; Kwon, J.; Chung, J. H.; Park, K. Y.; Nam, M. H. Nontargeted metabolite profiling in compatible pathogen-inoculated tobacco (*Nicotiana tabacum* L. cv. Wisconsin 38) using UPLC-Q-TOF/MS. *J. Agric. Food Chem.* **2012**, *60*, 11015–11028, doi:10.1021/jf303702j.
10. Shih, C. H.; Siu, S. O.; Ng, R.; Wong, E.; Chiu, L. C. M.; Chu, I. K.; Lo, C. Quantitative analysis of anticancer 3-deoxyanthocyanidins in infected sorghum seedlings. *J. Agric. Food Chem.* **2007**, *55*, 254–259, doi:10.1021/jf062516t.
11. Clifford, M. N.; Kirkpatrick, J.; Kuhnert, N.; Roozendaal, H.; Salgado, P. R. LC-MS^n analysis of the *cis* isomers of chlorogenic acids. *Food Chem.* **2008**, *106*, 379–385.
12. Mareya, C.R.; Tugizimana, F.; Piater, L.A.; Madala, N.E.; Steenkamp, P.A.; Dubery, I.A. Untargeted metabolomics reveal defensome-related metabolic reprogramming in *Sorghum bicolor* against infection by *Burkholderia andropogonis*. *Metabolites* **2019**, *9*, 8; https://doi:10.3390/metabo9010008
13. Tugizimana, F.; Djami-Tchatchou, A.T.; Steenkamp, P.A.; Piater, L.A.; Dubery, I.A. Metabolomic analysis of defence-related reprogramming in *Sorghum bicolor* in response to *Colletotrichum sublineolum* infection reveals a functional metabolic web of phenylpropanoid and flavonoid pathways. *Front. Plant Sci.* **2019**, *9*, 1840; doi.org/10.3389/fpls.2018.01840.
14. Carlson, R.; Tugizimana, F.; Steenkamp, P.A.; Dubery, I.A.; Labuschagne, N. Differential metabolic reprogramming in *Paenibacillus alvei*-primed *Sorghum bicolor* seedlings in response to *Fusarium pseudograminearum* infection. *Metabolites* **2019**, *9, 150; doi:10.3390/metabo9070150.*
